# CCR4 promotes metastasis via ERK/NF-κB/MMP13 pathway and acts downstream of TNF-α in colorectal cancer

**DOI:** 10.18632/oncotarget.10256

**Published:** 2016-06-23

**Authors:** Baochi Ou, Jingkun Zhao, Shaopei Guan, Hao Feng, Xiongzhi Wangpu, Congcong Zhu, Yaping Zong, Junjun Ma, Jing Sun, Xiaohui Shen, Minhua Zheng, Aiguo Lu

**Affiliations:** ^1^ Department of General Surgery, Ruijin Hospital, Shanghai Jiao Tong University School of Medicine, Shanghai, 200025, China; ^2^ Shanghai Minimally Invasive Surgery Center, Ruijin Hospital, Shanghai Jiao Tong University School of Medicine, Shanghai, 200025, China; ^3^ Shanghai Institute of Digestive Surgery, Ruijin Hospital, Shanghai Jiao Tong University School of Medicine, Shanghai, 200025, China; ^4^ Department of General, Visceral, Transplantation, Vascular and Thoracic Surgery, Hospital of the University of Munich, 81377 Munich, Germany

**Keywords:** CCR4, colorectal cancer, metastasis, MMP13, TNF-α

## Abstract

Chemokines and chemokine receptors are causally involved in the metastasis of human malignancies. As a crucial chemokine receptor for mediating immune homeostasis, however, the role of CCR4 in colorectal cancer (CRC) remains unknown. In this study, we found that high expression of CCR4 in CRC tissues was correlated with shorter overall survival and disease free survival. *In vitro* and *in vivo* experiments revealed that silencing CCR4 attenuated the invasion and metastasis of CRC cells, whereas ectopic overexpression of CCR4 contributed to the forced metastasis of these cells. We further demonstrated that matrix metalloproteinase 13 (MMP13) played an important role in CCR4-mediated cancer cell invasion, which is up-regulated by ERK/NF-κB signaling. Positive correlation between CCR4 and MMP13 expression was also observed in CRC tissues. Moreover, our investigations showed that the level of CCR4 could be induced by TNF-α dependent of NF-κB activation in CRC cells. CCR4 might be implicated in TNF-α-regulated cancer cells metastasis. Combination of CCR4 and TNF-α is a more powerful prognostic marker for CRC patients. These findings suggest that CCR4 facilitates metastasis through ERK/NF-κB/MMP13 signaling and acts as a downstream target of TNF-α. CCR4 inhibition may be a promising therapeutic option for suppressing CRC metastasis.

## INTRODUCTION

Colorectal cancer (CRC) is one of the major health problems worldwide. Although the majority of primary tumors can be resected surgically, prognosis is still poor for patients with advanced stage due to high rates of tumor recurrence and metastasis [1]. In order to give rise to successful metastasis, cancer cells need to achieve several important steps including invasion into surrounding tissues, detachment from the primary sites, survival in circulation, dissemination to distant organs, and formation of micrometastases [2]. To date, a variety of proteins and signaling pathways have been found to be closely related with cancer metastasis [3]. However, detailed mechanisms responsible for CRC metastasis remain poorly understood.

Chemokines belong to a superfamily of small molecules whose effects are regulated by binding to G-protein-coupled receptors [4]. They have been implicated in organ-specific metastasis of cancer by attracting tumor cells with matching chemokine receptors to specific sites. Mounting evidences have reported the crucial function of chemokines and their receptors in cancer. For example, CXCR4 has been found to be involved with metastasis in more than 20 different cancers [5–7]. In CRC, the study by Kawada et al. revealed that CXCR3 could promote cancer metastasis to lymph nodes [8]. Our previous studies showed that CCL19 suppressed tumorigenesis and metastasis, and the expression of CCL19 was related to the prognosis of patients with colorectal cancer [9, 10].

As an important chemokine receptor, CCR4 is characterized by mediating immune homeostasis and is selectively expressed on regulatory T cells and Th2 cells [11]. The functionof CCR4 has been previously elucidated in both hematologic malignancies and solid tumors such as gastric cancer [12], breast cancer [13] and lung cancer [14]. Moreover, the study by Al-haidari et al. suggested that CCR4 might participate in CCL17-induced migration of colon cancer cells. Nevertheless, little is known about the role of CCR4 itself in the progression of colorectal cancer. In the present study, we investigated CCR4 expression in CRC tissues and its correlation with a variety of clinicopathologic factors. Effects of CCR4 on cell invasion and metastasis were explored by both *in vitro* and *in vivo* assays. Underlying molecular mechanisms of CCR4 in CRC metastasis were also revealed. Furthermore, our findings suggest that CCR4 acts downstream of TNF-α and might play a role in TNF-α-mediated cancer cells metastasis.

## RESULTS

### Aberrant overexpression of CCR4 in colorectal cancer tissues

**Figure 1:**
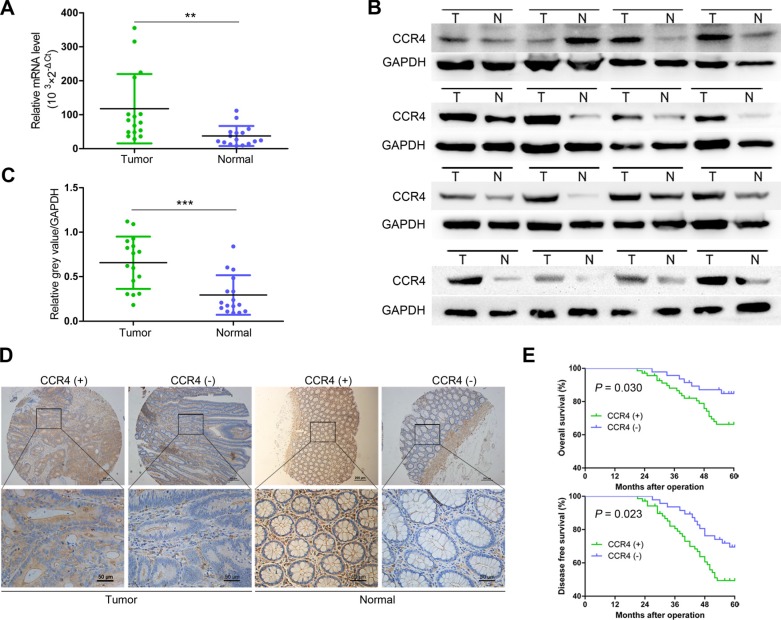
Expression of CCR4 and its clinical significance in CRC patients (**A**) qRT-PCR analysis showing CCR4 expression in 16 paired CRC samples that were randomly selected from the 116 CRC cases, by random numbers generated with SAS software. (**B**) Representative western blot images of CCR4 expression in 16 paired CRC samples. (**C**) Quantification of relative grey value of bands compared with GAPDH, as detected by western blot. (**D**) CCR4 expression level in tumor tissues and the paired normal tissues was evaluated by immunohistochemical staining with tissue microarray. (**E**) CRC patients with positive expression of CCR4 presented with worse overall survival, and disease free survival compared with that of negative expression of CCR4. Data represent the mean ± SD and are representative of three independent experiments. *P < 0.05, **P < 0.01, ***P < 0.001.

To investigate the function of CCR4 in CRC development, we first evaluated the mRNA expression in CRC specimens. As shown in Figure 1A, CCR4 transcript levels were significantly increased in CRC samples compared to paired normal tissues from 16 patients (*P* < 0.01, Figure 1A), which was further confirmed at the protein level by western blot (Figure 1B and Figure 1C). In addition, we examined the expression of the CCR4 protein by immunohistochemical (IHC) staining in a retrospective cohort of 116 pairs of cancerous and matched noncancerous tissue samples from CRC patients (Figure 1D). In these cases, CCR4 positive expression was detected in 69 (59.5%) of the tumor tissues, whereas only 53 (45.7%) of the adjacent normal specimens showed a positive CCR4 signal (*P* = 0.035, Table 1).

**Table 1:** Relationship between CCR4 expression level and clinicopathologic variables in 116 CRC patients

Variable	Case (*n* = 116)	CCR4 expression		*P* value
		Positive	Negative	
Tissues				0.035
Carcinoma	116	69	47	
Normal tissues	116	53	63	
Gender				
Male	68	41	27	0.832
Female	48	28	20	
Age				0.979
≤ 65	52	31	21	
>65	64	38	26	
Location				
Left hemicolon	12	7	5	0.340
Right hemicolon	33	19	14	
Sigmoid colon	24	11	13	
Rectum	47	32	15	
Tumor size(cm)				0.036
≤ 4 × 3	53	26	27	
> 4 × 3	63	43	20	
Tumor histology				0.921
Tubular	98	59	39	
Mucinous	16	9	7	
Papillary	2	1	1	
Extent of invasion				0.020
T1 + T2	31	13	18	
T3 + T4	85	56	29	
Lymphatic metastasis				0.018
N0	56	26	30	
N1 + 2	60	43	17	
Metastasis				0.113
M0	105	60	45	
M1	11	9	2	
TNM stage				0.030
I + II	55	27	28	
III + IV	61	42	19	
CEA level				0.674
< 5.0	89	52	37	
≥ 5.0	27	17	10	

### Clinicopathologic significance of CCR4 expression in CRC patients

To explore the clinicopathologic significance of CCR4 in CRC, we compared several clinical and pathologic factors with CCR4 expression (Table 1). A significant association was observed between the CCR4 negative group and positive group in tumor size (*P* = 0.036), invasion depth (*P* = 0.020), lymphatic metastasis (*P* = 0.018), and TNM stage (*P* = 0.030). Kaplan–Meier survival curves showed a strong correlation between CCR4 level and poorer 5-year overall survival (OS) (*P* = 0.030, Figure 1E). Moreover, CCR4-positive patients also had a shorter disease-free survival (DFS) (*P* = 0.023, Figure 1E). The data of multivariate analysis by Cox proportional hazards models suggested that, however, CCR4 expression was not an independent prognostic risk factor (data not shown).

### CCR4 promotes CRC cells metastasis *in vitro* and *in vivo*

**Figure 2:**
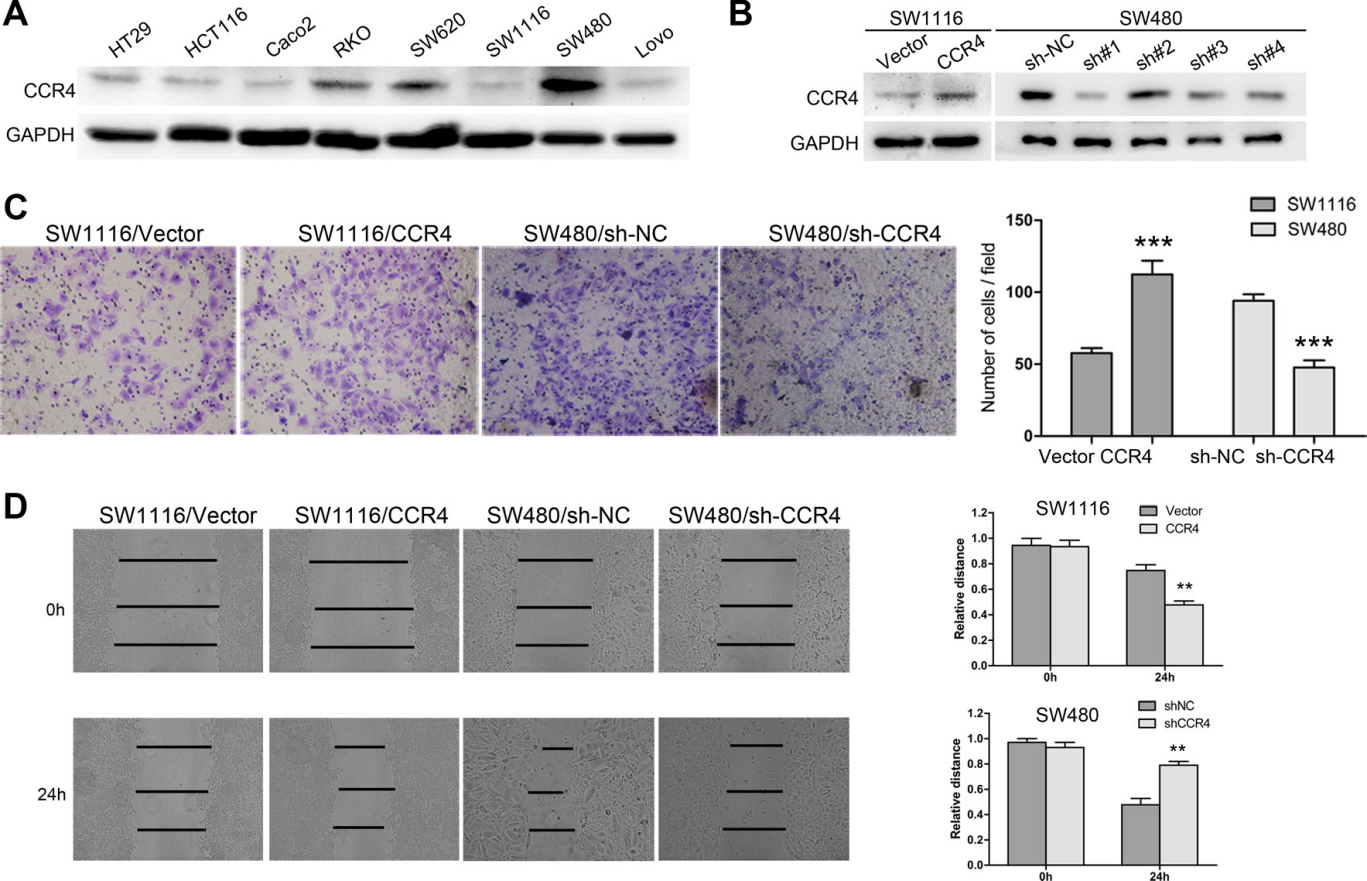
CCR4 plays a crucial role in the metastasis of SW1116 and SW480 cells (**A**) CCR4 expression in eight CRC cell lines detected by western blot. (**B**) SW1116 and SW480 cells transfected with pcDNA-CCR4 and sh-CCR4, respectively, were subject to western blot. (**C**) Invasive behavior was evaluated using matrigel invasion assays after overexpression or knockdown of CCR4 in SW1116 or SW480 (magnification, ×200). (**D**) The migratory capacity of SW1116/CCR4 and SW480/sh-CCR4 cells was analyzed by wound-healing assay.

**Figure 3:**
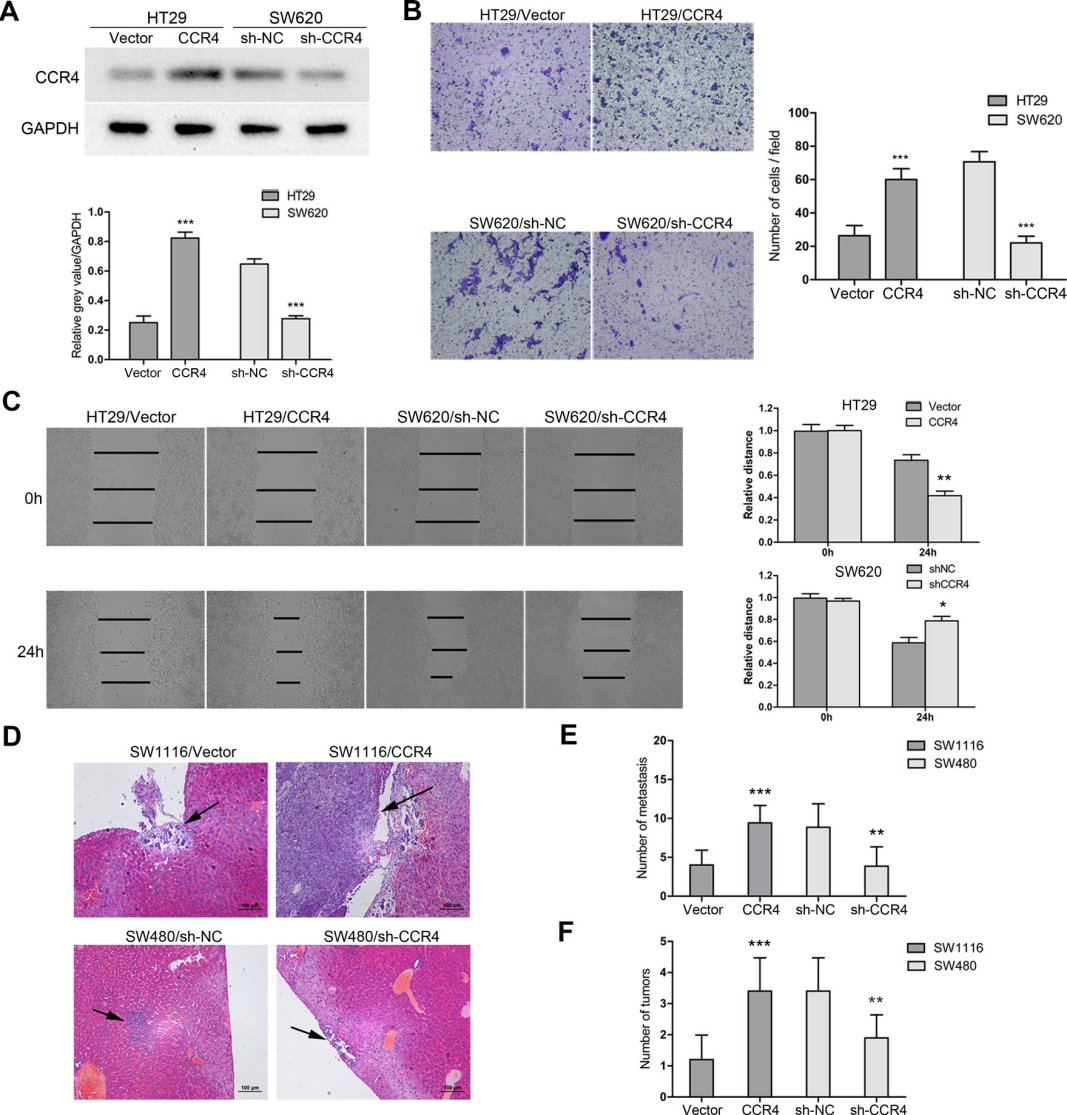
CCR4 facilitates CRC cells metastasis *in vitro* and *in vivo* (**A**) HT29 and SW620 cells transfected with pcDNA-CCR4 and sh-CCR4, respectively, were tested by western blot. (**B**) Matrigel invasion assays were performed to assess invasive ability of HT29/CCR4 and SW620/sh-CCR4 (magnification, ×200). (**C**) Wound-healing assay shows a significant increase or decrease in healing rate of the scramble wound in HT29/CCR4 and SW620/sh-CCR4, respectively. (**D**) Representative images of hematoxylin & eosin staining of liver tissue sections. Black arrows indicated liver metastasis. (**E**) Number of metastasis in the liver. (**F**) Number of tumors on the surface of liver given orthotopic implantation of xenograft. Data represent the mean ± SD and are representative of three independent experiments. *P < 0.05, **P < 0.01, ***P < 0.001.

In order to explore roles of CCR4 in CRC cells, we first detected its level in eight CRC cell lines. The results showed that CCR4 highly expressed in SW480 and SW620, and lowly expressed in SW1116 and HT29 (Figure 2A). Therefore, we generated a SW1116 cell line ectopically overexpressing CCR4 and employed lentivirus-mediated shRNA to knock down CCR4 in SW480, respectively. The effect of overexpression and knockdown was confirmed by western blotting (Figure 2B). Transwell assays showed that increased level of CCR4 promoted invasive abilities of SW1116 cells (Vector group: 57.67 ± 3.51, CCR4 group: 112.33 ± 9.61), whereas invasive potential was dramatically impaired in SW480/sh-CCR4 cells compared to sh-NC cells (sh-NC group: 94.00 ± 4.58, sh-CCR4 group: 47.67 ± 5.03) (Figure 2C). Additionally, wound healing assays showed that the distance between wound edges of SW1116/CCR4 cells was markedly longer than those of SW1116/Vector cells. A shorter distance was also observed in wound healing of SW480/sh-CCR4 cells compared with control group (Figure 2D). Moreover, these findings were further confirmed in SW620 and HT29 cells (Figure 3A–3C).

Next, we established two models of metastatic CRC, including liver metastasis and *in situ* transplantation, to investigate roles of CCR4 in CRC cells metastasis *in vivo*. The representative images of metastatic lesions in the liver were indicated in Figure 3D. Compared to control cells, overexpression of CCR4 in SW1116 cells increased liver metastases dramatically (Vector group: 4.00 ± 1.91, CCR4 group: 9.43 ± 2.23). On the contrary, depletion of CCR4 resulted in significant decrease of metastatic foci in SW480 cells (sh-NC group: 8.86 ± 3.02, sh-CCR4 group: 3.86 ± 2.48) (Figure 3E). Moreover, the results from orthotopic model showed that CCR4 played a positive role in metastasis of CRC cells (Vector: 1.20 ± 0.79 versus CCR4: 3.40 ± 1.07, sh-NC: 3.37 ± 1.07 versus sh-CCR4: 1.90 ± 0.74, Figure 3F). Collectively, these results suggested functional significance of CCR4 expression in CRC cells metastasis.

### MMP13 is responsible for CCR4-mediated CRC cells invasion

**Figure 4:**
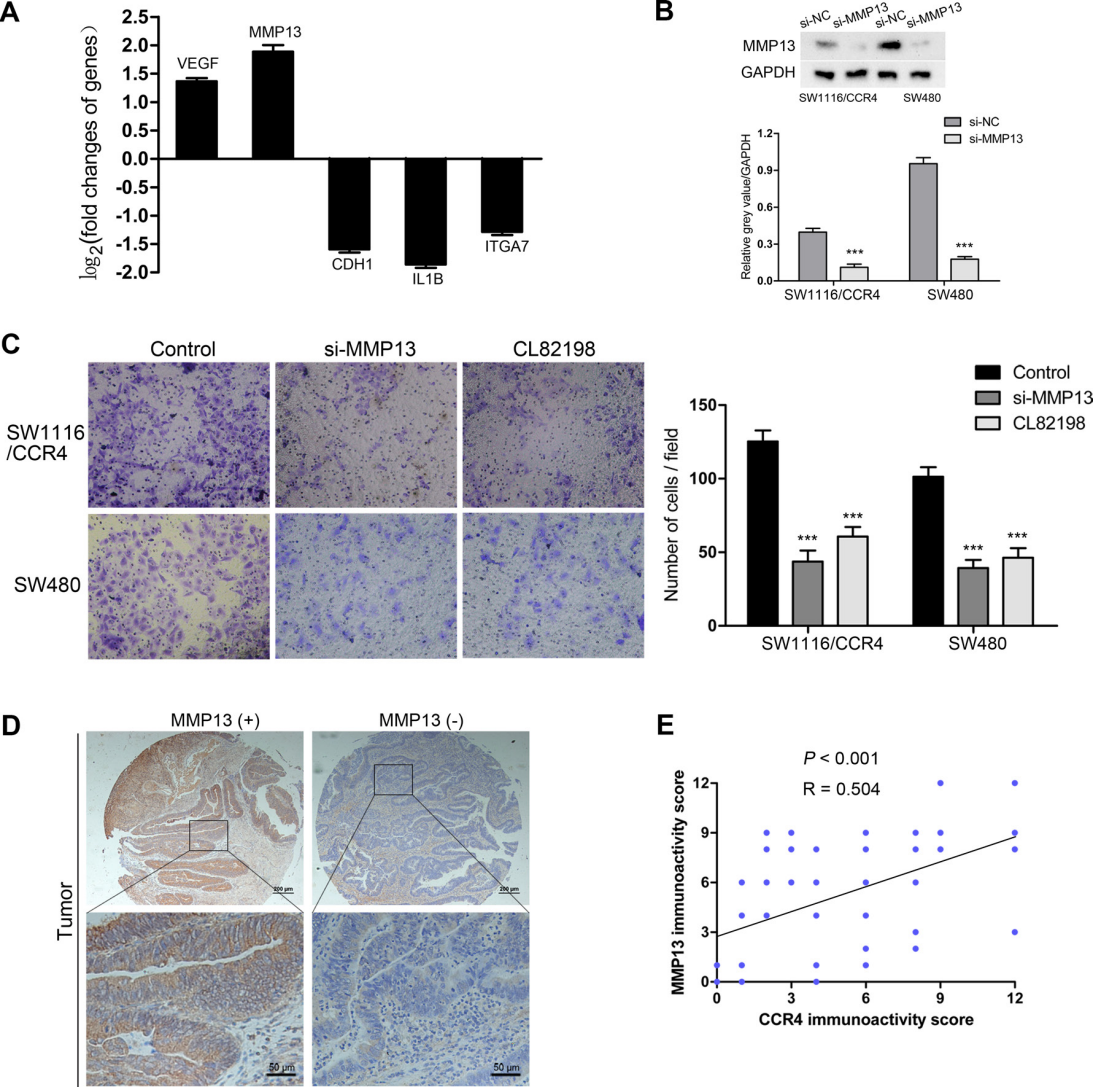
MMP13 plays a crucial role in CRC cells invasion mediated by CCR4 (**A**) Five metastasis-related genes (VEGF, MMP13, CDH1, IL1B, ITGA7) showed a more than 2-fold mRNA differential expression in PCR array. (**B**) The effect of MMP13 knockdown in SW1116/CCR4 and SW480 cells, detected by western blot. Densitometry represents the expression of the proteins relative to GAPDH. (**C**) Results of invasion assays showed the inhibitory roles of si-MMP13 or CL82198 on SW1116/CCR4 and SW480 cells (magnification, ×200). (**D**) Representative images of MMP13 staining in the cohort of 116 CRC tissues. (**E**) Expression correlation of CCR4 and MMP13 was analyzed in 116 CRC patients using IHC.

To further explore the molecular mechanisms that CCR4 involved in CRC cells invasion, we analyzed metastasis-related genes for SW1116/CCR4 cells and SW1116/Vector cells using a Tumor Metastasis PCR Array. Among 84 metastasis-related genes examined, two up-regulated (VEGF, MMP13) and three down-regulated (CDH1, IL1B, and ITGA7) metastasis-related genes were observed, which had a more than 2-fold change in mRNA levels (Figure 4A). MMP13 was selected for further investigation because it has the largest up-regulated alteration in SW1116/CCR4 cells compared with control group cells. To investigate whether MMP13 is crucial for CCR4-mediated cell invasion, RNA interference was used to knockdown MMP13 expression in SW1116/CCR4 and SW480 cells, whose effects were confirmed by western blot analysis (Figure 4B). After si-MMP13 treatment, the increased capacities of invasion induced by CCR4 overexpression were dramatically abolished in SW1116/CCR4 cells. Similarly, number of invasive cells also decreased remarkably after MMP13 knockdown in SW480 (Figure 4C). Moreover, when cells treated with CL82198 (a MMP13 inhibitor), invasive abilities of SW1116/CCR4 and SW480 cells were also significantly reduced (Figure 4C).

We then analyzed MMP13 expression in the TMA containing 116 paired CRC tissues (Representative images: Figure 4D). As expected, MMP13 was highly expressed in tumor samples compared to normal samples (69.8% versus 30.2%, *P* < 0.001, Table 2). Furthermore, our results indicated a positive correlation between CCR4 expression and MMP13 (Pearson's correlation, *r* = 0.504, *P* < 0.001, Figure 4E).

**Table 2:** The expression level of MMP13 and TNF-αin 116 CRC specimens

Variable	Tissues (*n* = 116)		*P* value
	Carcinoma	Normal tissues	
MMP13 expression			*P* < 0.001
Positive	81	47	
Negative	35	69	
TNF-α expression			*P* = 0.002
Positive	77	54	
Negative	39	62	

### MMP13 is up-regulated via Erk1/2/NF-κB pathway

**Figure 5:**
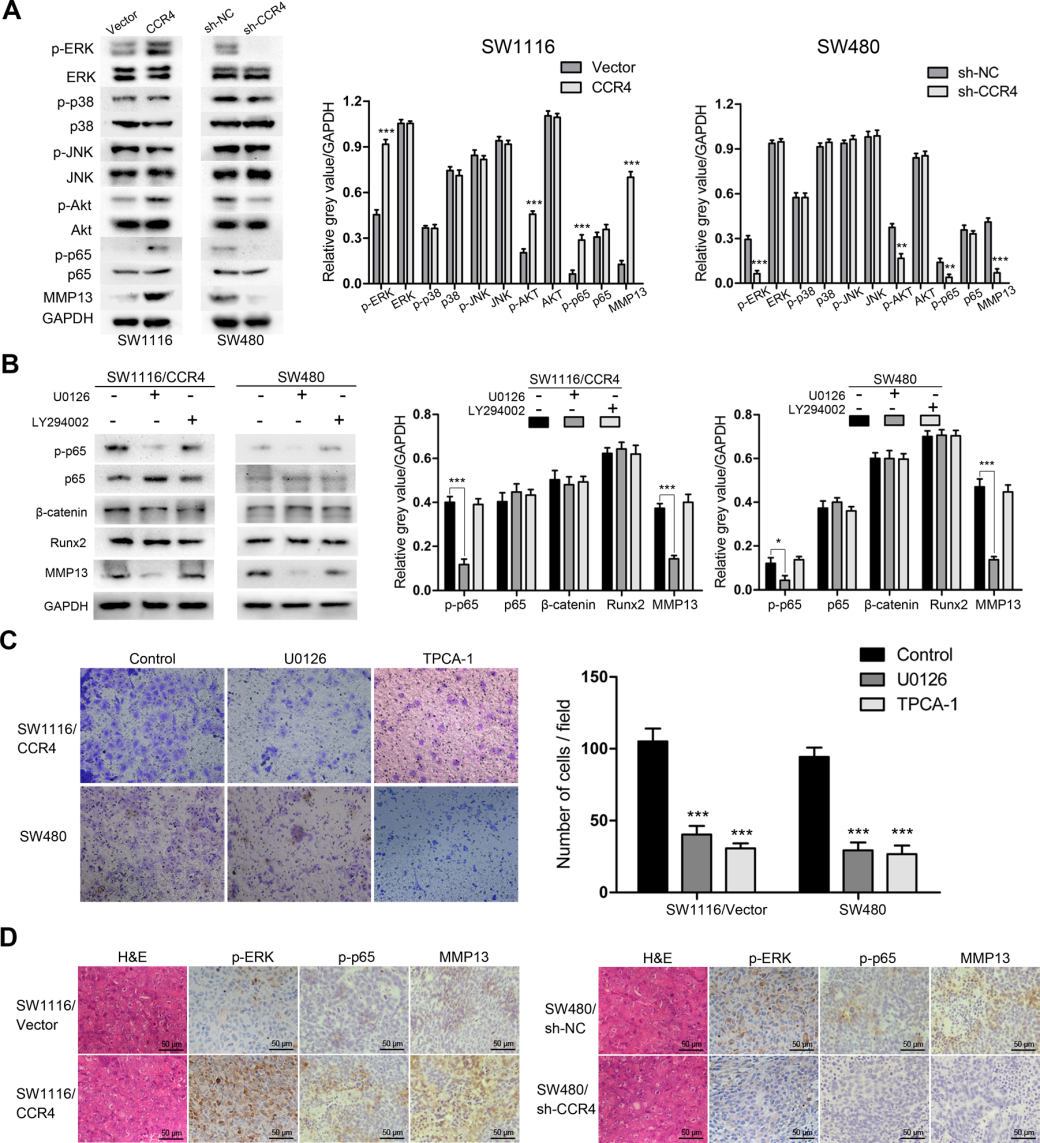
CCR4 up-regulates MMP13 expression through Erk1/2/ NF-κB activation (**A**) p-ERK, p-p38, p-JNK, p-Akt, p-p65 and MMP13 expressions were determined by western blot analysis. Densitometry represents the expression of the proteins relative to GAPDH. (**B**) p-p65, β-catenin, Runx2, and MMP13 expressions were analyzed using western blot. Densitometry represents the expression of the proteins relative to GAPDH. (**C**) Results of invasion assays showed the inhibitory roles of U0126 or TCPA-1 on SW1116/CCR4 and SW480 cells (magnification, ×200). (**D**) Representative images of IHC staining of p-ERK, p-p65 and MMP13 in the CRC tissues of orthotopic implantation model in nude mice. Scare bars = 50 μm. *P < 0.05, **P < 0.01, ***P < 0.001.

Chemokines have been shown to work by activating some signaling pathways, including mitogen-activated protein kinase (MAPK) and phosphatidylinositol 3-kinase (PI3K)/protein kinase B (Akt). Thus, we conducted western blot analysis to elucidate the possible signal mechanisms responsible for CCR4-mediated up-regulation of MMP13. The results indicated that both of p-ERK and p-Akt were activated in SW1116/CCR4 and SW480 cells (Figure 5A). Next, we used the inhibitors of ERK (U0126) and PI3K (LY294002) to preincubate these cells and found that the MMP13 was induced via the activation of ERK but not Akt (Figure 5B). To further investigate downstream targets, the levels of possible molecules that have been reported to involve with the up-regulation of MMP13 were tested, including NF-κB, β-catenin and Runx2 [19–21]. The results showed that blockade of ERK pathway significantly inhibited the level of activated p65 in SW1116/CCR4 and SW480 cells (Figure 5B). Moreover, inhibition of ERK or NF-κB signaling pathway by U0126 or TPCA-1 dramatically suppressed invasion of SW1116-CCR4 and SW480 cells (Figure 5C). To verify the regulatory pathway *in vivo*, the association among p-ERK, p-p65, MMP13 was explored in tissues from orthotopic implantation model. As expected, phosphorylation of ERK, p65, and MMP13 expression were markedly increased in metastatic lesion of SW1116/CCR4 cells as compared to them in lesion of SW1116/Vector cells, and were obviously decreased in metastatic lesion of SW480/sh-CCR4 cells as compared to control group (Figure 5D). Taken together, CCR4-meidated up-regulation of MMP13 was dependent on ERK/ NF-κB pathway in CRC cells.

### CCR4 acts as a downstream target of TNF-α

**Figure 6:**
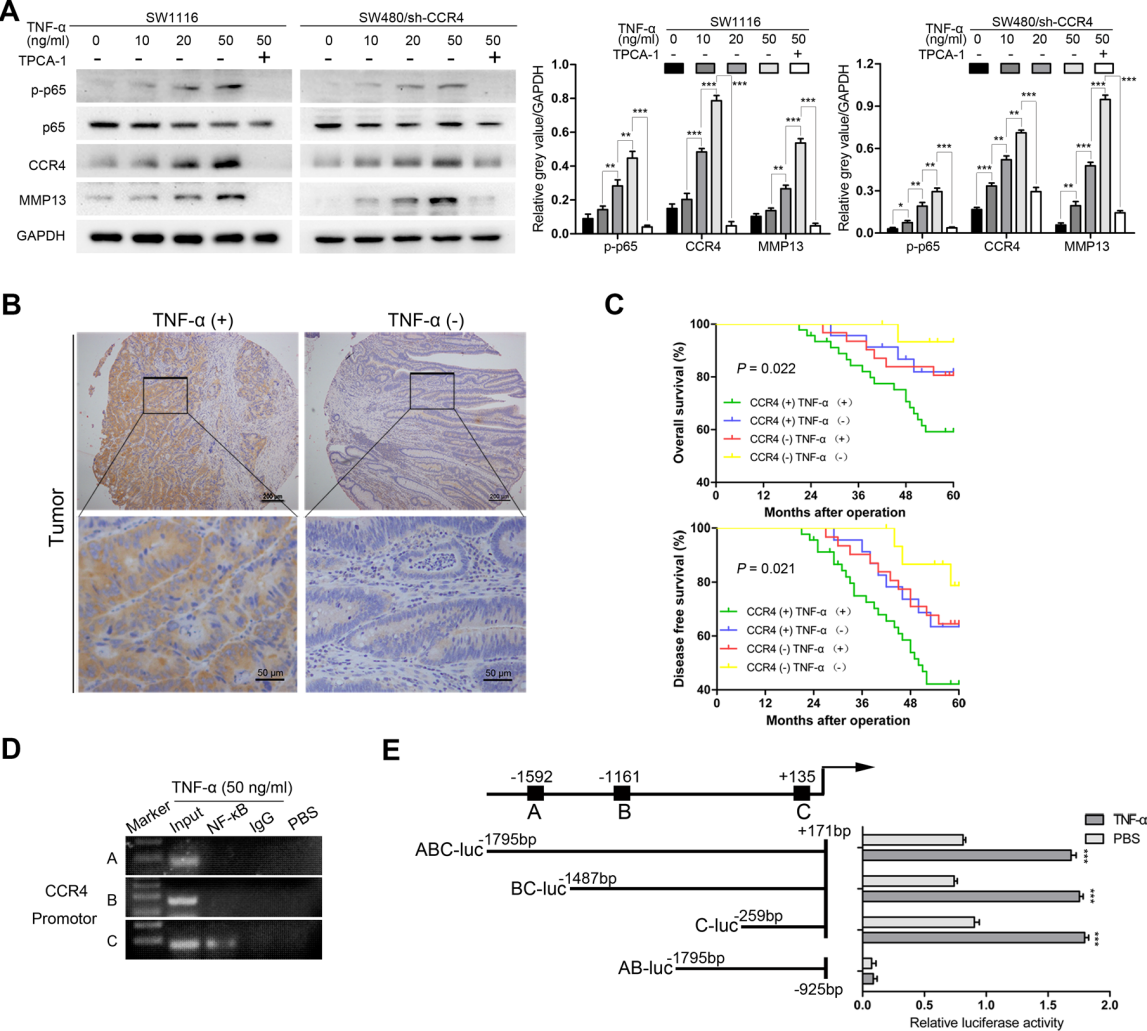
CCR4 could be induced by the cytokine TNF-α (**A**) Cells were incubated with different doses of TNF-α for 12 h, and p-p65, CCR4 or MMP13 expression is examined by western blot. (**B**) Representative images of IHC staining of TNF-α in tissue microarray. (**C**) Prognostic values of CCR4 combined with TNF-α. (**D**) DNA fragments pulled down with NF-κB antibody from SW1116 cells treated with TNF-α (50 ng/ml) or PBS were amplified by PCR. (**E**) The promoter activity was evaluated by transfection with different CCR4 luciferase expression vectors. Cells were treated with TNF-α (50 ng/ml) or PBS for 12 h.

Previously, a variety of evidences revealed that pro-inflammatory cytokines, such as TNF-α, IL-1β, TGF-β, could induce metastasis of tumor cells via up-regulating chemokine receptors [22–24]. These findings point to a critical role of chemokine receptors as a link between inflammation and cancer. Notably, the study by Yang et al. demonstrated that CCR4 could be activated by cytokine TNF-α in gastric cancer [25]. Thus, we sought to determine whether TNF-α contributed to the expression of CCR4 as well in CRC cells. The cells expressing low level of CCR4 (SW1116 and SW480/sh-CCR4) were treated with different concentrations of rh-TNF-α, interestingly, we found that the expression of CCR4 increased in a dose-dependent manner after 12 h incubation (Figure 6A). Moreover, a dose-dependent up-regulation of MMP13 was observed in TNF-α-stimulated cells (Figure 6A), suggesting the chemokine receptor CCR4 might be involved in TNF-α-induced expression of MMP13 in CRC cells. Collectively, CCR4 may play an important role in TNF-α-mediated cancer cells metastasis.

In addition, the expression of TNF-α was examined in the TMA and a significant higher level was found in tumor specimens than that in normal specimens (*P* = 0.002, Table 2, Figure 6B). CRC patients whose tumors with high levels of both CCR4 and TNF-α exhibited worst prognoses (OS: *P* = 0.022, DFS: *P* = 0.021, Figure 6C). However, multivariate analysis indicated that combination of CCR4 and TNF-α did not represent an independent prognostic factor for CRC patients (data not shown).

It is well-known that TNF-α activates signal transduction pathways that could culminate in activation of the transcription of NF-κB, we thus tried to investigate whether CCR4 functions as a NF-κB target. The results showed that inhibiting the activation of NF-κB abrogated TNF-α-stimulated CCR4 expression (Figure 6A). Next, the upstream sequences of CCR4, ranging from −2000 to +200 bp with respect to the transcription start site were analyzed. Three potential binding sites were identified within the CCR4 promoter region, which were located in −1597/−1587, −1166/−1156, and +130/+140. We then performed chromatin immunoprecipitation (ChIP) assays with SW1116 cancer cells incubated with TNF-α (50 ng/ml) or PBS. As illustrated in Figure 6D, only primer-C of CCR4 produced strong PCR products, which suggested that NF-κB formed complexes with the C binding site in the CCR4 promoter. Luciferase reporter assays also suggested that the potential C binding site in the CCR4 promoter is required for transactivation of the downstream gene upon TNF-α stimulation (Figure 6E).

## DISCUSSION

Great efforts have been made to explore the molecular basis of invasion and metastasis for cancer cells in the past decade, nonetheless, the critical mechanisms that truly contribute to metastasis of colorectal cancer still remain incompletely understood. Growing evidences indicate that chemokines and their receptors are involved in the complex processes of cancer metastasis [7]. In this study, our investigations showed that the chemokine receptor CCR4 was significantly associated with poor prognosis of CRC patients. A remarkable correlation between expression of CCR4 and tumor size, invasion depth, or lympthatic metastasis was found, indicating that cancer cells with high level of CCR4 have more invasive phenotype. Although CCR4 did not represent an independent risk factor for patients with colorectal cancer, these results still showed a critical role of CCR4 in colorectal cancer.

It has been reported that CCR4 overexpresion can enhance metastatic potential in breast cancer and lung cancer [13, 26]. Olkhanud et al. found that breast cancer lung metastasis required CCR4 expression and regulatory T cells [26]. Moreover, primary tumors can induce the production of CCR4 ligands in the lungs of mice, which enable CCR4 positive tumor cells to migrate more easily [26]. The data we presented herein revealed that overexpression of CCR4 significantly facilitated invasion of CRC cells *in vitro* and enhanced distant liver metastasis *in vivo*. In contrast, silencing of CCR4 reduced the invasive capacity of CRC cells *in vitro* and *in vivo*. Our above findings suggest the chemokine receptor CCR4 may play a crucial role in CRC cells metastasis.

To investigate the potential molecular mechanism involving CCR4-induced cells invasiveness, we profiled differentially expressed metastasis-related genes with a Tumor Metastasis PCR array.and identified MMP13 as a candidate target. MMP13 could be produced by various types of cancer cells and serves as a critical regulator of metastatic process in human malignancies [18, 19]. It has been reported that MMP13 is related to cancer aggressiveness in hepatocellular carcinoma [27]. Stromal MMP13 mediates tumor microenvironment and promotes lung metastasis of breast cancer [28]. In the present study, expression level of MMP13 was found to be decreased under condition of CCR4 knockdown, while overexpression of CCR4 increased the level of MMP13 in tumor cells. Depletion or inactivation of MMP13 significantly suppressed CCR4-mediated invasion of CRC cells. These data provide new clues on the underlying mechanism that CCR4 might promote colorectal cancer cell invasion by up-regulation of MMP13. Next, the potential signal pathways implicated in the regulation of MMP13 expression were explored and the results showed that both ERK and PI3K-Akt pathways possibly participated in this process. When treated with small chemical inhibitors against key factors of pathways (U0126 and LY294002), we found that MMP13 expression was inhibited by the blockaded of ERK signaling in tumor cells. Recently, ERK/MMP pathway was shown to facilitate epithelial-to-mesenchymal transition in colorectal cancer [29]. The study by Liang et al. [30] indicated that interleukin-1-induced MMP9 expression required the activation of ERK signaling. Our data herein found that ERK might play an important role in the expression of MMP13 and invasiveness of human CRC cells.

Growing evidences show that many growth factors stimulate the expression of MMP genes via signal transductions that converge to activate transcription factors. To date, multiple transcription factor consensus binding motifs have been observed in the MMP13 promoter, including NF-κB, β-catenin and Runx2 [19–21]. In the present study, we demonstrated that NF-κB but not β-catenin or Runx2, modulated the CCR4-mediated MMP13 activity in colorectal cancer cells, suggesting a crucial function of NF-κB in regulating invasiveness of tumor cells.

It is generally recognized that the cytokine TNF-α, produced by immune cells such as macrophages, T and B cells, exhibits both proinflammatory and immunoregulatory activity. Up-regulation of TNF-α has the capacity to increase the chemokine receptor CXCR4 levels, which promotes the invasiveness of gastric cancer cells [31]. In this study, we found a significant positive correlation between expression of CCR4 and TNF-α in CRC tissues by using IHC analysis. *In vitro* experiments also indicated that TNF-αcontributed to the elevation of CCR4 expression in CRC cells. Moreover, the chemokine receptor CCR4 might be involved in TNF-α-induced expression of MMP13 in CRC cells, suggesting the drugs targeting CCR4 may provide a clue for antagonizing TNF-α-regulated cancer cells metastasis. These findings indicate that CCR4 is not constitutively expressed in CRC cells but may be up-regulated in tumor microenvironment. Considering that the immune microenvironment of tumor cells is so complex, however, there may be other factors could contribute the expression of CCR4.

Previous studies elucidated that both NF-κB and TNF-α played important roles in connecting inflammation to tumor progression [32]. As a heterodimeric transcription activator, NF-κB consists of the DNA binding subunit p50 and the transactivation subunit p65 [33]. The activation of NF-κB allows itself to translocate into the nucleus and transcriptionally activate target genes. In this study, we found that TNF-α increased the expression level of CCR4 in NF-κB-dependent manner. Suppressing the activation of NF-κB completely abrogates TNF-α -induced CCR4 expression. Furthermore, NF-κB binds to promoter region of CCR4 and contributes to its expression.

In conclusion, our data unravel a novel mechanism that the chemokine receptor CCR4 facilitates metastasis of CRC cells by activating ERK/ NF-κB /MMP13 pathway. Overexpression of CCR4 is correlated with poor survival of CRC patients. Moreover, CCR4 could be induced by the cytokine TNF-α in NF-κB-dependent manner and might be involved in TNF-α-induced cancer cells invasiveness. These findings support that targeting CCR4 may be a promising strategy for suppressing metastasis in CRC.

## MATERIALS AND METHODS

### Cell culture and reagents

Eight human CRC cell lines were purchased from American Type Culture Collection (ATCC, VA, USA) and were preserved by Shanghai Digestive Surgery Institute. SW480, SW620 and SW1116 are cultured in Leibovitz's L-15medium supplemented with 10% fetal calf serum. HCT116, HT29, Caco2, RKO and LoVo were maintained in RPMI-1640 medium with the same components. All of these cells were incubated at 37°C and 5% CO2. U0126 (MEK inhibitor), LY294002 (PI3K-Akt inhibitor), TPCA-1 (IKK inhibitor) and CL82198 (MMP13 activity inhibitor) were purchased from Selleckchem (Houston, USA) and used in accordance with manufacturer's instructions. TNF-α was purchased from R&D systems.

### Generation of gene overexpressing and knockdown stable cells

pGLV-GFP-CCR4 lentiviral vector and four shRNA plasmids targeting different regions of CCR4 mRNA were purchased from Genepharma (Shanghai, China). Lentivirus particles were transfected into the CRC cells in the presence of polybrene and selected for 2 weeks using 5 μg/ml puromycin. CCR4 sequences targeted were as follows: shRNA1: 5′-GGT TCT GGA CAC CTT ACA ACA-3′; shRNA2: 5′-GCA CCT TTG AAA GAT ACT TGG-3′; shRNA3: 5′-GGG AGA AAT TTC GCA AGT ACA-3′; shRNA4: 5′-GCA GTC CAC CAT GGA TGG ATC ATG A-3′. Plasmids were transfected into cells with Lipofectamine 2000 (Invitrogen) and stable transfected cells were selected by G418. The plasmid shRNA1 proved the strongest efficiency and was used for further research.

### Transient transfection

MMP13 siRNA and control siRNA were purchased from Genepharma. The cells were transfected with siRNA using Lipofectamine 2000 according to the manufacturer's instructions. After 6 h, the medium was changed to normal medium and cells were cultured for further 48 h.

### Patients and immunohistochemical analysis

The collection of specimens from 116 patients was authorized by Ethics Committee of Ruijin Hospital used after obtaining informed consent. These specimens embedded with paraffin were made into tissue microarray (TMA) by Shanghai Outdo Biotech Company, as previously described [34]. The staining of TMA was conducted according to the manufacturer's protocol (Immunostain SP kit, DakoCytomation, USA). Antibodies used for immunohistochemical analysis included antibody against CCR4 (ab1669), TNF-α (Sigma) and MMP13 (Abcam). The results of immunostaining were determined by staining intensity and the number of positive cells (staining intensity: negative = 0, weak = 1, moderate = 2, strong = 3; and the percentage of cells stained: 0 = 0–1%, 1 = 1–5%, 2 = 6–29%, 3 = 30–59%, 4= 60–100%). Immunohistochemical score was independently determined by two pathologists who were blinded to patient characteristics.

### Real-time quantitative reverse transcription-PCR (qRT-PCR)

Total RNA was isolated from cell lines and tissues using Trizol (Invitrogen) according to the manufacturers' instructions. cDNA was synthesized by using reverse transcription kit (Invitrogen, CA). Quantitative polymerase chain reaction (PCR) was performed by using SYBR Green PCR Master Mix (Applied Biosystems, UK). Primers for CCR4 were as followed: forward 5′-AGA AGG CAT CAA GGC ATT TGG-3′ and reverse 5′-ACA CAT CAG TCA TGG ACC TGA G-3′. Relative mRNA expression was calculated by comparative Ct method and GAPDH was used as the control. All experiments were done in triplicate.

### Tumor metastasis PCR array analysis

The Human Tumor Metastasis PCR Array (SABiosciences, USA), which includes 84 genes known to be involved in metastasis, was used to profile SW1116/CCR4 and the control group cells according to the protocols. Briefly, cDNA constructed from RNA using RT2 First Strand Kit (Qiagen) was combined with specific RT^2^ qPCR Master Mix. Then, equal aliquots of this mixture were added to each well of the PCR Array plate containg genes-pecific primers. The reaction and data collection were performed on Applied Biosystems^®^ 7500 Real-Time PCR Systems.

### Immunoblotting

Western blot analysis was performed as previously described [35]. 100 ug of protein was separated by 10% SDS-PAGE gel and transferred to PVDF membranes. The membranes were blocked with 5% non-fat milk for 2 h and then were incubated at 4°C overnight with primary antibodies. The primary antibodies for CCR4, p-p65, p65, β-catenin and MMP13 were purchased from Abcam, and antibodies against ERK (4695), p-ERK (4377), JNK (9252), p-JNK (4668), p38 (9212), p-p38 (4631), Akt (4691), p-Akt (13038), and Runx2 (8486) were purchased from Cell Signaling (Danvers, MA). Horseradish peroxidase-conjugated secondary antibodies were used and the protein bands were visualized by an Odyssey scanner (LI-COR Biosciences).

### Wound healing assay and matrigel invasion assay

Cells were cultured in serum-free medium for 24 h and wounded with pipette tips. Fresh medium was then replaced. 24 h later, the wound closing procedure was observed and photographed. The invasion assays were performed as previously reported [36]. Briefly, 200 μL serum-free medium containing 2 × 10^5^ cells was added into the upper chamber and 600μL medium with 10% serum was added into the lower chamber. The chamber coated with diluted Matrigel (BD) was cultured in 37°C, 5% CO2 condition for 24 h and fixed with methanol. Cells that invaded to the bottom of the membrane were stained with 1% crystal violet and photographed under microscope. Three independent experiments were conducted for the same conditions.

### Luciferase assay

CCR4 promoter fragments were amplified from human genomic DNA, and were inserted into pGL3-Basic vector. SW1116 cells were cotransfected with pGL3-CCR4-promotor constructs and treated with 50 ng/ml TNF-α or PBS. 24 hours later, luciferase activity was examined using the Dual Luciferase Assay (Promega) following the manufacturer's instructions.

### Chromatin immunoprecipitation (ChIP)

The ChIP assay was performed according to the protocol of chromatin immunoprecipitation kit (Millipore). Protein and DNA was cross-linked in 1% formaldehyde, extracted by SDS lysis buffer, and sheared by sonication. The supernatants were immunoprecipitated with NF-κB antibodies (Santa Cruz, sc-166588) or an isotype control IgG for 2 hours. After purification of precipitated DNA, PCR was conducted. Primers used for PCR were listed in Supplementary Table 1.

### Mice

CRC cells were injected into the spleens of mice under anesthesia (*n* = 8 for each group). The spleen was removed after injection to avoid splenic tumor formation, so that metastatic lesions developed only in the liver. The animals were sacrificed 30 days later and the numbers of tumor colonies in the livers were measured. For *in situ* transplantation model, cells were inoculated subcutaneously into the left armpit of nude mice (*n* = 4). Xenograft tumors were then removed and transplanted into the left lateral lobe of liver in other mice (*n* = 10 for each group). Four weeks later, metastatic tumors on the surface of liver were examined. All experimental protocols were performed according to the Guide for the Care and Use Laboratory Animals of Ruijin Hospital, Shanghai Jiaotong University School of Medicine. Four-week-old male BALB/C nude mice (Institute of Zoology, China Academy of Sciences) were used in experiments.

### Statistics

All tests were performed by SAS. Quantitative variables were analyzed by Student *t* tests. Correlations between CCR4 and MMP13, TNF-α expression were analyzed by Spearman test. Log-rank test in Kaplan-Meier method and Cox regression model was used to assess patients' survival outcome and prognostic factors. *P* < 0.05 was considered to be significant.

## SUPPLEMENTARY MATERIALS


