# Correction: CCR4 promotes metastasis via ERK/NF-κB/MMP13 pathway and acts downstream of TNF-α in colorectal cancer

**DOI:** 10.18632/oncotarget.18562

**Published:** 2017-06-19

**Authors:** Baochi Ou, Jingkun Zhao, Shaopei Guan, Hao Feng, Xiongzhi Wangpu, Congcong Zhu, Yaping Zong, Junjun Ma, Jing Sun, Xiaohui Shen, Minhua Zheng, Aiguo Lu

**Present:** A reference for this paper was accidentally omitted from the article.

**Correct:** The missing reference appears below.

15. Al-haidari AA, Syk I, Jirström K, Thorlacius H. CCR4 mediates CCL17(TARC)-induced migration of human colon cancer cells via RhoA/Rho-kinase signaling. Int J Colorectal Dis. 2013; 28:1479-1487.

Original article: Oncotarget. 2016; 7:47637-47649. doi: 10.18632/oncotarget.10256

