# Supplementary material for: CCR4 promotes metastasis via ERK/NF-κB/MMP13 pathway and acts downstream of TNF-α in colorectal cancer
Source: Oncotarget. 2016 Jun 23;7(30):47637–49. doi: 10.18632/oncotarget.10256 (PMC5216967; doi:10.18632/oncotarget.10256)
Supplement: Supplementary file 1 [file oncotarget-07-47637-s001.pdf]

CCR4 promotes metastasis via ERK/NF-κB/MMP13 pathway and acts downstream of TNF-α in colorectal cancer

Supplementary Materials

Supplementary Table S1: Primers for PCR in chromatin immunoprecipitation

Primer		sequence	position	product
Primer A	F	TTGCTGTGGTGGGCTGTC	–1863 bp	80 bp
	R	GGGAGTTACTACTGGCTTT	–1492 bp	
Primer B	F	GAGCCTGAGTTGAGTGCCT	–1487 bp	165 bp
	R	TCCAGATGCTTTCCCTGT	–1047 bp	
Primer C	F	GTTGGGTGAGGGAGATAAC	–92 bp	78 bp
	R	TCAGCCAGGTCCAGAAAG	+162 bp	
